# Is the open access citation advantage real? A systematic review of the citation of open access and subscription-based articles

**DOI:** 10.1371/journal.pone.0253129

**Published:** 2021-06-23

**Authors:** Allison Langham-Putrow, Caitlin Bakker, Amy Riegelman

**Affiliations:** 1 University of Minnesota Libraries, University of Minnesota, Minneapolis, MN, United States of America; 2 University of Minnesota Health Sciences Libraries, Minneapolis MN, United States of America; Universitat de Barcelona, SPAIN

## Abstract

**Aims:**

Over the last two decades, the existence of an open access citation advantage (OACA)—increased citation of articles made available open access (OA)—has been the topic of much discussion. While there has been substantial research to address this question, findings have been contradictory and inconclusive. We conducted a systematic review to compare studies of citations to OA and non-OA articles.

**Methods:**

A systematic search of 17 databases attempted to capture all relevant studies authored since 2001. The protocol was registered in Open Science Framework. We included studies with a direct comparison between OA and non-OA items and reported article-level citation as an outcome. Both randomized and non-randomized studies were included. No limitations were placed on study design, language, or publication type.

**Results:**

A total of 5,744 items were retrieved. Ultimately, 134 items were identified for inclusion. 64 studies (47.8%) confirmed the existence of OACA, while 37 (27.6%) found that it did not exist, 32 (23.9%) found OACA only in subsets of their sample, and 1 study (0.8%) was inconclusive. Studies with a focus on multiple disciplines were significantly positively associated with finding that OACA exists in subsets, and are less associated with finding that OACA did not exist. In the critical appraisal of the included studies, 3 were found to have an overall low risk of bias. Of these, one found that an OACA existed, one found that it did not, and one found that an OACA occurred in subsets.

**Conclusions:**

As seen through the large number of studies identified for this review, OACA is a topic of continuing interest. Quality and heterogeneity of the component studies pose challenges for generalization. The results suggest the need for reporting guidelines for bibliometrics studies.

## Introduction

Scholarly publishing relies on the gift economy of academia in which authors provide content for free and editors and peer reviewers donate their time to review that content [1, 2]. Prior to the development of the internet, the costs of printing, shipping, and other operations were offset by user and institutional subscriptions. As publishing became more commercialized in the second half of the 20th century, journal subscription prices began increasing, outpacing both inflation and university budgets [3–7]. Despite the shift from print to electronic, prices have only risen.

Various modes of open access (OA) publishing have emerged over the last 30 years to replace or augment subscription-based (“toll-access”) publishing. Green OA refers to materials that are made openly available through archiving in an OA repository or other legally permissible venue. Depending on journal policies and the decision-making of the depositor, a green OA version could be an early version of a manuscript or a copy-edited, post-peer review version. Historically, Gold OA was used to refer to “OA delivered by journals, regardless of the journal’s business model” [8 p. 52]. Gold OA can be further broken into gold journals (fully OA journals, which may or may not charge article processing charges (APCs)) and hybrid journals (which charge authors an APC to make their article open in a subscription journal).

Over the last 20 years, researchers and publishers have introduced a multitude of categories of OA beyond green and gold. These include "diamond" or "platinum" OA (i.e., OA in the place of publication but without an APC charged [9, 10]), “bronze” OA (i.e., articles that publishers make available to readers for free, but do not have an OA license [11]), and "grey" OA (i.e., authors make their content available via upload to an academic social network or personal website, potentially without consideration of previously signed copyright transfer agreements). Others have used the term “black OA” to mean access facilitated by the posting of materials to pirate sites, such as SciHub [12]. It is important to note that this type of access does not necessarily involve the consent or participation of the author or publisher; as such, it is questionable whether this is truly a mode of OA. Because terminology has developed over time, we refer only to green OA versus gold OA, as these are the most broadly adopted categories of OA.

The potential for OA publication to increase citations was first articulated in an empirical study of computer science in 2001 [13]. Since 2001, there have been many studies both supporting and refuting the existence of an open access citation advantage (OACA). This nomenclature is consistently used in the literature, even in those studies reporting negative or null findings.

Although many relevant primary studies and some secondary studies exist on this topic, the influence of OA on citation remains unclear, particularly when considering the range of disciplines, data sources, publishing models, and other contextual factors. In a critical review, Davis and Walters [14] found a notable increase in the number of downloads for articles published OA but no clear evidence of OACA. Additionally, Davis and Walters found that studies finding citation advantages for OA failed to “adequately control for confounding variables” [14 p. 208]. In a narrative review, Turk [15] noted that OACA studies varied greatly in terms of data collection platforms used (e.g., Scopus, Google Scholar), differing publication types (e.g., journal articles, conference papers), and different disciplines analyzed, which potentially influenced the variety of findings.

In an update to the literature review by Davis and Walters [14], Lewis [16] concluded that only a “few of the authors…actually claim causation”, and instead pointed to the insight gained by examining correlation between OA and a citation advantage (CA). Lewis argued that more research is needed "to prove a causal relationship between OA and CA" [16 p. 59]. Like Turk [15] and others, Lewis noted that differences existed in studies targeting certain disciplines, and future research across all disciplines would "provide a wider array of evidence for the occurrence of field-specific OACA and therefore of a more widespread OACA" [16 p. 59].

A 2007 critical review by Craig et al. looked at three non-exclusive postulates when examining citation differences between both OA and non-OA articles: that the advantage is due to 1) OA status of the article; 2) selection bias (i.e., authors select their best works to share openly), and 3) early view effects (i.e., the extra time that an article is available) [17]. They pointed to studies in which controlling for one postulate (e.g., early view) revealed that citation counts can be explained by another postulate (e.g., selection bias) and advised that more rigorous methods were needed in future studies to look at causation [17].

Hua et al. [18] published a narrative review specific to OA concepts in dentistry in which they go beyond OACA and venture into other topics such as research waste of inaccessible research. Regarding OACA, they looked at nine studies across many disciplines and found variation of methods and materials, but ultimately the citation advantage ranged from -5% to 83% [18]. Turk [19] published an overview on OA, pertaining only to medical articles, looking at OA factors in which studies of possible OACA mostly found a citation advantage, but articles reporting randomized controlled trial (RCT) results revealed no OACA. Turk [19] indicated that other factors may influence a citation advantage for OA such as discipline, impact factor of journals studied, and the early view effect and selection bias Craig noted in 2007 [17].

Although these reviews provide valuable context, they were not as comprehensive and the methods were not transparently reported to the level of rigor needed to ensure reproducibility or replicability. The present study is unique in that there are no other existing systematic reviews or meta-analyses comparing citations of OA and non-OA articles. We address this need and critically appraise the existing studies to better understand the totality of the evidence while also identifying future areas of research. These findings could be of value to researchers when making evidence-based decisions about which mode of publishing—OA or otherwise—may most closely align with some of their objectives when choosing a publication venue.

## Methods

In accordance with best practices for systematic reviews, a combination of controlled vocabulary and natural language searching was used to comprehensively capture relevant studies (S1 Checklist). The search included iterations of terms for OA and citation advantage. A primary database search strategy was developed in Ovid Medline and then translated to the following databases reflecting a range of disciplines: Library & Information Science Source (LISS), Library Information Science & Technology Abstracts (LISTA), ERIC, Academic Search Premier, and Business Source Premier via EBSCO; PubMed; Embase, CAB Abstracts and PsycINFO via Ovid; Scopus; Web of Science Core Collection; Compendex; Sociological Abstracts, EconLit, and Dissertations & Theses Global via ProQuest; and SHARE. A full search strategy of our primary database is available in S1 Appendix.

As indicated in our inclusion and exclusion criteria, this project targeted only studies published after 2001, which is the date of the earliest known article on citation advantage of OA [13]. By study, we refer to those items that were retrieved, screened, and included in this project. By articles, we refer to the subject of those studies: the OA and non-OA materials for which citation counts were being gathered. The searches were initially conducted in July 2019 and were rerun in November 2020. We did not include any filters to limit language, study design, or publication type. To ensure that no potentially relevant items were overlooked, hand searching of reference lists was undertaken. In cases where limited or unclear data were available, authors were contacted for clarification and to seek additional information. Any non-English language content was translated into English for screening and, if applicable, extraction and assessment. The protocol was registered in Open Science Framework (osf.io/p2a7q).

Two screeners independently reviewed every title and abstract, applying previously determined inclusion and exclusion criteria. In order to be included, studies needed to contain a direct comparison between a sample of OA articles and non-OA articles, with citation counts as an outcome, and have been published since January 2001. We excluded studies that did not report citation count data, such as narrative reviews, editorials, or opinion pieces; did not include citation counts as an outcome; and/or did not directly compare OA versus non-OA articles. For example, a study that reported on citation counts for a set of OA articles against a different set of OA articles would be excluded; a study that compared citations to gold OA articles with citations to subscription articles would be included even if the authors did not take into account the possible overlap of green OA articles within either set.

**Fig 1:**
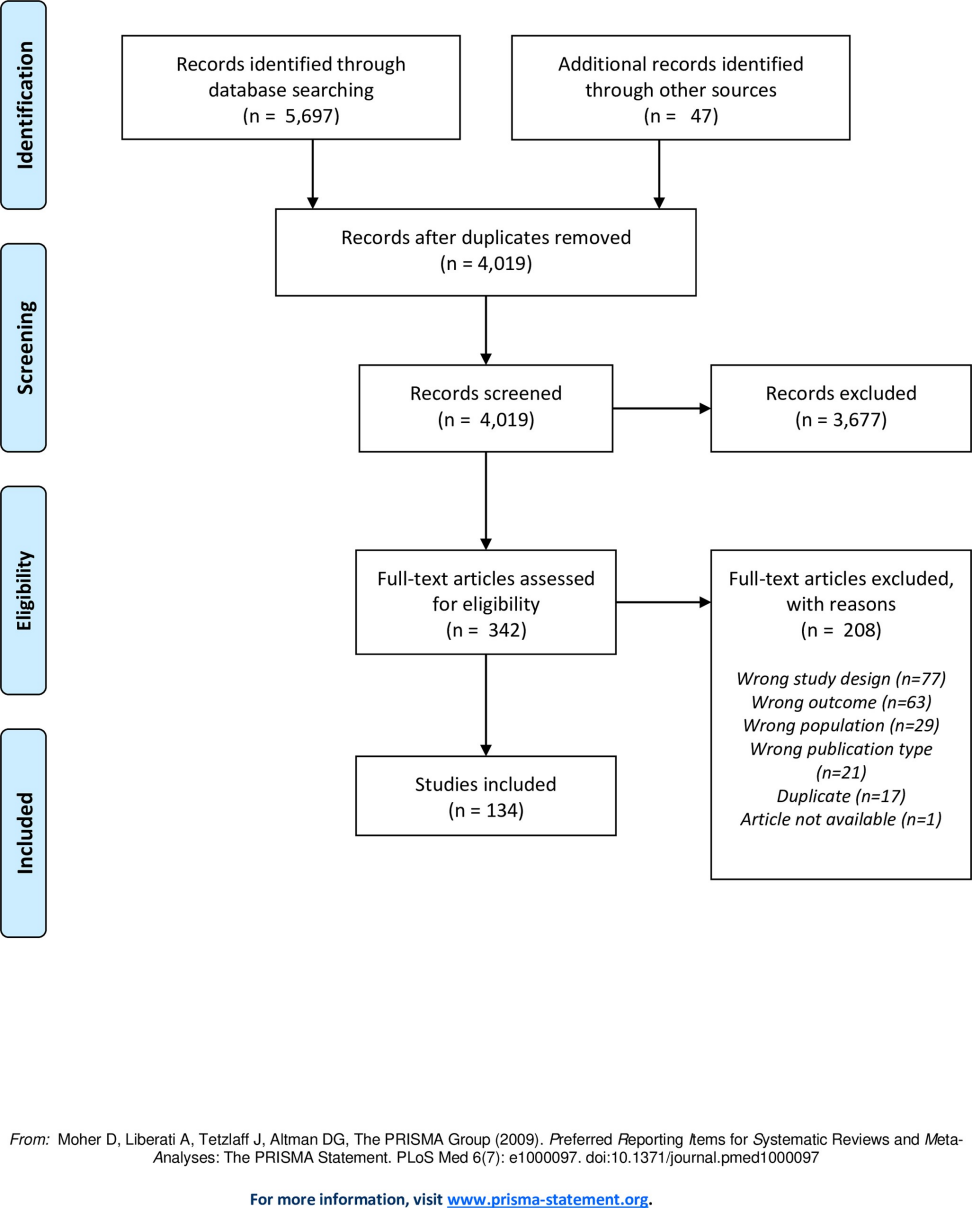
PRISMA flowchart. PRISMA flowchart showing number of studies at each step of the selection process.

Screening was completed using Rayyan, a web application that facilitates independent screening [20]. Where there were discrepancies, the conflicts were resolved via discussion or by a third person tie-breaker when necessary. Full-text screening was also completed by two independent screeners, and reasons for exclusion were recorded and are reported in Fig 1.

The data extraction form was developed and piloted by three researchers. Data elements extracted included information about the study, including the publication type of the study, as well as the type of metrics used to measure citations, mode of OA (green, gold, or a combination), and overall findings of the study. With respect to categorization of the mode of OA, we relied on the study authors’ reporting of OA status. As noted in the introduction, more nuanced definitions of OA exist. However, these broader categories are more commonly used and have been in effect for longer, thus making them more applicable for the purposes of this study. As hybrid and gold OA were sometimes conflated within the included studies, these categories are combined in this project. Similarly, some authors used “green OA” to describe whether a version of the article was findable through a search engine (i.e., available in an OA repository or academic social network but not through a pirate site).

We extracted data elements describing the metrics used and the findings of the study. These elements were categorized into larger groupings for further analysis. Metrics were grouped into mean, median, total, and other, representing the most common methods of measurement, while overall findings were grouped as OACA exists, OACA does not exist, or OACA exists in subsets of the data. We extracted the disciplinary subject of each study, and subsequently mapped these subjects to the six major subject codes outlined by the Organisation for Economic Cooperation and Development (OECD)’s Field of Science and Technology Classification [21]. Use of OECD overcomes challenges in using database or publisher-specific resources, such as taxonomies from Elsevier or Clarivate, while providing a framework applicable to a broad range of disciplines and document types. The disciplinary subject of each study was defined as the disciplinary subject of the articles included in the study, rather than the discipline of the journal that published the study. Where the subject matter reflected more than one classification, the item was marked as “multiple disciplines.” Data extraction was completed independently by two researchers, as was risk of bias assessment. The Evidence-based Librarianship (EBL) critical appraisal tool developed by Glynn [22] was used, as this tool was developed for library and information science literature and therefore would be the most broadly applicable to the literature included in this study. Where there were any discrepancies in the data extracted or the risk of bias assessment, these discrepancies were resolved through discussion or by a third party when necessary.

Following data extraction, heterogeneity of the studies was assessed using the I^2^ statistic to determine if quantitative synthesis would be appropriate. I^2^ was calculated using RevMan 5.3 [23]. An I^2^ of 90% was found among studies with sufficient data for pooling. As such, the studies were found to be significantly heterogeneous and quantitative synthesis was deemed inappropriate. Although we were not able to pool the data in a meta-analysis, chi-squared tests were conducted to analyze the associations between findings, as categorical variables, and their associations with characteristics of studies. These tests were performed using R 3.6.0 [24].

## Results

5,697 results were retrieved through database searching, and 47 additional items were retrieved through handsearching. Once duplicates were removed, 4,019 items were subject to title-abstract screening. Of these, 3,677 were excluded, leaving 342 items for full-text assessment. 208 items were excluded at the full-text screening phase, resulting in 134 studies that were ultimately included in this analysis [11, 25–157].

Of the 134 items, more fully described in Table 1, 132 were non-randomized studies while 2 were randomized. The studies most frequently addressed multiple disciplines (n = 45, 33.6%), followed by Medical and Health Sciences (n = 36, 26.9%), Natural Sciences (n = 22, 16.4%), and Social Sciences (n = 21, 15.7%). Detailed information on included studies is available in S1 Dataset.

**Table 1:** Overview of included studies.

Study Design		
	Randomized	2 (1.5%)
	Non-Randomized	132 (98.5%)
Discipline (OECD classification)		
	Agricultural Sciences	4 (3.0%)
	Engineering and Technology	2 (1.5%)
	Medical and Health Sciences	36 (26.9%)
	Multiple disciplines	45 (33.6%)
	Natural Sciences	22 (16.4%)
	Social Sciences	21 (15.7%)
	Unspecified	4 (3.0%)
Data Sources		
	Web of Science/Clarivate	82 (61.2%)
	Scopus/Elsevier	38 (28.4%)
	Google Scholar	29 (21.6%)
	Other	19 (14.2%)
OA Modes Included		
	Green	25 (18.7%)
	Gold	53 (39.6%)
	Green & Gold	33 (24.6%)
	Unsure/Unspecified	23 (17.2%)
Metrics Used		
	Mean Citations Per Article	88 (65.7%)
	Median Citations Per Article	33 (24.6%)
	Cumulative Citations	21 (15.7%)
	Other Metrics	50 (37.3%)
Finding		
	OA Citation Advantage Exists	64 (47.8%)
	OA Citation Advantage Exists in Subsets	32 (23.9%)
	OA Citation Advantage Doesn’t Exist	37 (27.6%)
	Inconclusive	1 (0.8%)

The studies used a range of data sources. Most frequently, data were retrieved from Clarivate/Web of Science (n = 82, 61.2%), followed by Scopus (n = 38, 28.4%) and Google Scholar (n = 29, 21.6%), including Google Scholar data accessed through Publish or Perish [158]. 19 (14.2%) studies used other sources of data, and 24 (17.9%) studies used more than one source of data.

The majority of the studies (n = 64, 47.8%) found that there was OACA while 27.6% (n = 37) found that it does not exist. 23.9% (n = 32) of studies found that, while there was no overall OACA, there was an advantage in subsets, such as for specific journals, certain periods of time, or subdisciplines. One study (0.8%) was inconclusive in its findings. These studies based their conclusions on a range of measures.

The characteristics of the 134 studies and their associated findings are described in Table 2.

**Table 2:** Study characteristics and findings.

	Does Open Access Citation Advantage Exist?				
	Yes (n = 64)	No (n = 37)	Sometimes (n = 32)	Inconclusive (n = 1)	
OA Mode(s) Included					p = 0.161
Green (n = 25)	18	5	2	0	
Gold (n = 53)	21	19	12	1	
Green & Gold (n = 33)	13	8	12	0	
Unclear (n = 23)	12	5	6	0	
Metric(s) Used to Compare Citation Counts					p = 0.861
Mean (n = 88)	45	20	22	1	
Median (n = 33)	19	7	7	0	
Total (n = 21)	8	7	6	0	
Other (n = 50)	21	16	12	1	
Discipline (OECD Classification)					p < 0.001
Agricultural Sciences (n = 4)	1	3	0	0	
Engineering and Technology (n = 2)	0	0	2	0	
Medical and Health Sciences (n = 36)	19	14	3	0	
Multiple disciplines (n = 45)	19	6	20	0	
Natural Sciences (n = 22)	10	8	4	0	
Social Sciences (n = 21)	14	3	3	1	
Unspecified (n = 4)	1	3	0	0	
Data Source					p = 0.663
Scopus/Elsevier (n = 38)	16	12	10	0	
Web of Science/Clarivate (n = 82)	39	21	22	0	
Google Scholar (n = 29)	16	6	6	1	
Other (n = 19)	10	4	5	0	
Risk of Bias					p = 0.959
High (Overall) (n = 131)	63	36	31	1	
Low (Overall) (n = 3)	1	1	1	0	
High (Population) (n = 134)	64	37	32	1	
Low (Population) (n = 0)	0	0	0	0	
High (Data Analysis) (n = 107)	51	28	28	0	
Low (Data Analysis) (n = 27)	13	9	4	1	
High (Study Design) (n = 91)	43	27	21	0	
Low (Study Design) (n = 43)	21	10	11	1	
High (Results) (n = 118)	55	32	30	1	
Low (Results) (n = 16)	9	5	2	0	
Publication Type					p = 0.807
Article (n = 107)	52	30	24	1	
Open (n = 32)	15	7	9	1	
Subscription/Hybrid (n = 75)	37	23	15	0	
Conference Presentation (n = 15)	7	3	5	0	
Working Paper/Preprint (n = 8)	2	4	2	0	
Thesis (n = 4)	3	0	1	0	

### Mode of OA

There was no statistically significant relationship between mode of OA and findings. Findings that OACA existed were most common regardless of mode of OA: 72% (18/25) of studies focused on green OA, 39.6% (21/53) of those focused on gold OA, 39.4% (13/33) of those focused on both green and gold OA, and 52.2% (12/23) of items that did not specify mode found that OACA existed. While 39.6% of studies focused on gold OA found OACA, gold studies account for 51.4% (19/37) of all studies that found no OACA existed.

### Discipline

We found a statistically significant relationship between discipline and OACA finding (X^2^(18,134) = 42.763,p < 0.001). Studies addressing multiple disciplines were positively associated with finding that OACA sometimes exists, and are less associated with finding that OACA did not exist. While not statistically significant associations, findings of the existence of OACA were more common in Social Sciences (66.7%, 14/21), Medical and Health Sciences (52.8%, 19/36), and Natural Sciences (45.5%, 10/22). In both Agricultural Sciences and studies that did not specify discipline, 25% of studies found that OACA existed, while OACA was not found in 75% of studies. Of the 32 studies that found OACA was present in subsets, 20 (62.5%) addressed multiple disciplines.

### Risk of bias

The EBL critical appraisal tool considers the possibility of bias in four domains: (1) population, (2) data collection, (3) study design, and (4) results [22]. Of the 134 studies assessed, 3 were found to have an overall low risk of bias. Of the three studies that had low overall risk of bias, one found that an OACA existed, one found that it did not, and one found that an OACA occurred in subsets.

**Fig 2:**
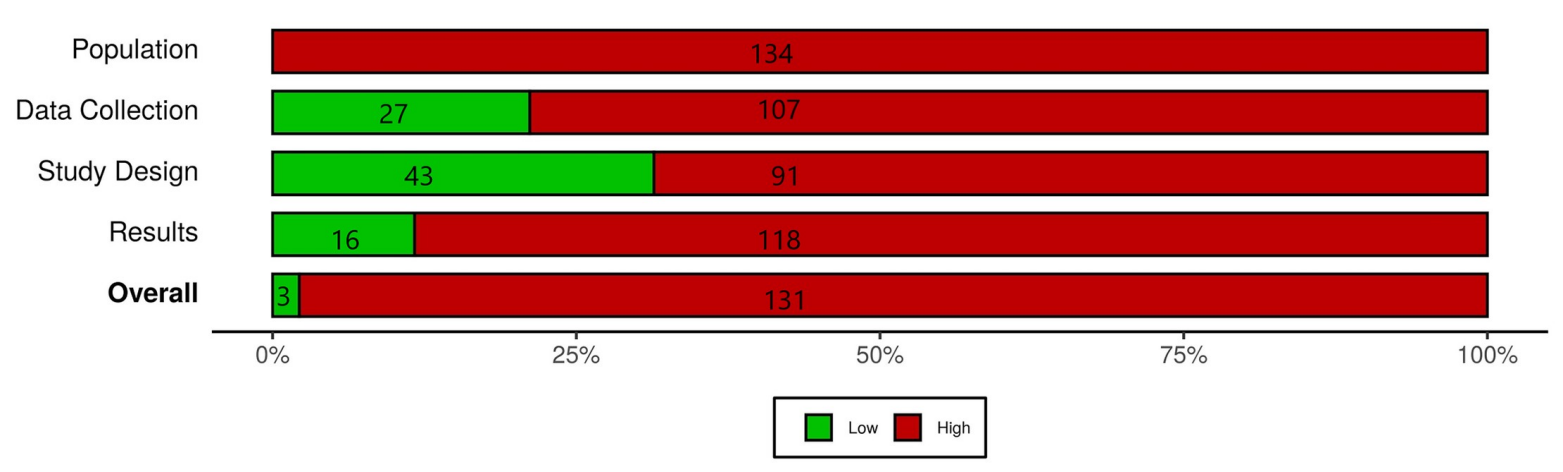
Risk of bias summary. Summary of overall risk of bias findings (low or high) and in the population, data collection, study design, and results domains.

All studies were found to have high risk of bias in the domain of population, 118 (88.1%) studies were found to have high risk of bias in the results domain, 107 (79.9%) were high risk of bias in data collection, and 91 (67.9%) had high risk of bias in study design. A summary of risk of bias assessment can be found in Fig 2, while an itemized risk of bias assessment for every component study can be found in S1 Dataset. There were no statistically significant associations between overall risk of bias and findings.

### Publication types

The majority of studies were published as journal articles (107/134), regardless of findings. Of those studies published as journal articles, the majority were published in subscription or hybrid journals (75/107). The remaining studies were made available through conference presentations (15/134), as working papers or preprints (8/134), or as theses or dissertations (4/134). There were no statistically significant relationships between publication type and findings of the studies.

## Discussion

The concept of OACA has been discussed since before the various declarations and definitions of OA [159–161]. In 2001, Lawrence reported that “free online availability substantially increases a paper’s impact.” [13 p. 521] As seen in our study, the topic has been the subject of numerous studies over the last 20 years.

Due to the heterogeneity of the sample of studies, a meta-analysis was not possible. We noted variations in the mode of OA, the types of publications, and the disciplines studied. There was also variation in the methods used and metrics reported. This suggests the need for reporting guidelines for bibliometric studies. At minimum, studies should report clearly on the mode of OA, the number of OA and non-OA articles, comparability of the data used, the time frame (citation window) and a justification for this choice, a common metric (e.g., mean and median citations per article), and confounding factors and how they are addressed.

With respect to the OA mode, studies should provide their definition of the mode of OA being considered. This may become even more necessary as researchers continue to create and implement new OA definitions. Additionally, if studying green OA, the study should assure that the articles in the non-OA population remain closed throughout the study and specify their exact working definition of “green” (e.g., whether articles found on academic social networks or author web pages are included). Similarly, studies exploring gold OA should consider whether articles in the OA sample are open throughout the entire citation window and the possibility that “non-OA” articles may have been openly available through other modes. The first consideration is complicated not only by potential delays in archiving an open copy, but also by new modes of OA. For example, with the introduction of bronze OA, publishers are free to move articles without an open license behind or in front of a paywall at any time and the status of an article at the time of data collection may differ from its previous status.

Although many studies reported metrics of mean citations per article and median citations per article, we found a wide variety of metrics studies reported. These included time-based metrics (e.g., citations per month since publication) and proportional metrics (e.g., difference in citations per paper, ratio of cited to non-cited articles). Other studies explored OACA by building linear regression models. The variations in methodology are not surprising, given the wide range of authors’ fields of research, including library and information studies, health sciences, computer science, and physics. OACA studies are published in bibliometric-focused journals, but also in discipline-specific journals. Disciplinary differences may explain some of the variation of methods employed. While a range of outcome measures can prove challenging, the consistent sharing of underlying data can help to overcome these barriers.

Very few (1.5%) of the studies in our sample were RCTs. There are notable challenges to conducting a randomized study on this topic. Beyond issues of funding, it may prove difficult to obtain consent from authors to randomize their articles to either condition. Instead, studies look retrospectively at populations of previously published articles. Nonrandomized studies face the challenge of ensuring that studies are truly comparable at baseline, allowing researchers to control for confounding factors including the potential "novelty" of the study, interest in the study, author reputation, and journal prestige [162]. Studies may also need to address the possibility of surreptitious cointerventions, such as toll access articles being made available through venues like ResearchGate, regardless of the journal’s OA policy. Although the prevalence of nonrandomized studies is understandable, these potential confounders pose challenges for identifying the implications of publishing decisions.

Out of the 134 studies included, 40 acknowledged the possibility of confounders, although not all 40 subsequently controlled for confounders in their analyses. Confounders noted included years since publication, Journal Impact Factor, number of authors, length of article, type of study (e.g., empirical or otherwise), prominence in search engine results, and the alphabetical position of the first author [77, 91, 136, 140, 142, 148, 149]. While examination of confounders is an important component of analysis, we found that this was inconsistently done and the confounders being considered varied significantly between studies.

We found high risk of bias in nearly 98% of the included studies (Fig 2). High risk of bias/low validity in the population domain was often due to a poorly described sample or use of too narrow a sample to support the conclusions drawn. Few studies provided justification for their sample size. We saw high risk of bias/low validity in data collection, often due to lack of justification for why a particular time frame was used (i.e., length of the citation window). Citations accrue over time, at rates that can vary across disciplines. OACA studies should take into account the citation patterns for the discipline being studied. High risk of bias/low validity in study design was often due to poor reporting of outcomes in relation to data collection.

Because of the limitations of the quality of the studies in our review, it is not possible to draw definitive conclusions and recommendations for authors deciding whether to make their work OA. We also recognize that venue choice is complex, and rarely driven by a single factor. Authors may be required to make work public or OA by funding agency mandates, or they may wish to do so to reach certain audiences. These decisions may be complicated, and arrived at through collaboration and discussion, and may be influenced by external factors such as career stage, departmental and organizational requirements, and disciplinary norms. In a survey of over 2,100 researchers at R1 institutions, respondents named journal reputation and quality, alignment between the article and journal scope, and the journal’s readership as the most important factors when selecting a journal, while the OA status of the journal was rated to be the least important consideration in the journal selection process, with 12% of respondents considering it to be very important and 18% of respondents considering it to be important [163]. Although the OA status of the journal seems to be relatively unimportant to many researchers, the potential impact of OA on citations remains an ongoing discussion.

One reason there is so much interest in whether OACA exists is due to the emphasis placed on citation metrics in retention, promotion, and tenure (RPT) decisions. Aksnes et al. [164] provide an overview of the use of citation metrics to evaluate the quality of research and whether citations accurately reflect quality or impact. The Scholarly Communications Lab (https://www.scholcommlab.ca/) has explored the use of citation metrics in RPT. Alperin et al. [165] analyzed 129 RPT documents from US and Canadian universities and found references to metrics in more than 70% of documents from research-intensive universities and nearly half of master’s colleges and universities.

However, citation metrics only measure the use of a study in the academic world. The goal of OA is to enable broader access to research; these uses may not be captured through citations. Scholars wish to publish in the venues that reach their audiences, for example, to reach practitioners who do not have institutional access to subscription resources. These uses may not result in subsequent citation in scholarly work, but are valuable nonetheless. Conversations around the need to and methods of acknowledging impact outside of an academic space have been ongoing, with organizations such as the European Commission noting that “[t]he exclusive use of bibliometric parameters as proxies for excellence in assessment by most funding agencies and universities/research organisations does not facilitate Open Science” [166 p. 8].

## Limitations and future work

There are limitations to our study. Most notably, we were unable to conduct a quantitative meta-analysis due to the heterogeneity of and the high risk of bias in our pool of studies. Our findings of high risk of bias for nearly all of the studies may be reflective of the lack of reporting guidelines. Reporting guidelines are “[a] checklist, flow diagram, or structured text to guide authors in reporting a specific type of research, developed using explicit methodology,” providing a list of the minimum information to be shared in each section of a paper [167]. Establishing reporting guidelines for bibliometrics studies and meta-research would be valuable in improving the completeness, clarity, and quality of studies in this area. Scholars would continue to have freedom regarding study design but have guidance on what details would need to be transparently reported. We also note that the EBL critical appraisal tool was designed for library and information research, but not specifically for bibliometric studies [22]. [To the best of our knowledge, there is no risk of bias assessment specifically designed for bibliometric studies, and the unique features of those studies. Development of such a tool, or modification of an existing tool for this purpose, could be of value. Although we did conduct searches across 17 databases, it is possible that relevant resources were not included. Non-English language content and publishers from outside of North America, the UK, and western Europe are underrepresented in scholarly databases, which may have resulted in potential omission in our study [105, 168].

The understanding and use of alternative metrics ("altmetrics”) has increased over the last 10 years [169]. Altmetrics measure the attention a work receives through metrics such as number of downloads, shares, or tweets on Twitter. These metrics have the potential to reflect use of research that would not be captured through scholarly citations, such as articles that are downloaded by healthcare providers and used in clinical care. Some of the studies in our sample addressed altmetrics (alternative metrics, such as number of downloads or views of an article). Studies included in our analysis occasionally considered altmetrics along with traditional citations (15%). An “open access altmetric advantage” could be evaluated through a review like this one.

As seen through the large number of studies identified for this review, OACA is a topic of high and continuing interest. In a now discontinued service, SPARC Europe previously tracked findings of studies measuring OACA [170]. Although this was a worthy effort, data collection ceased in 2015, the methods of identifying the included studies are not transparent or reproducible, and no risk of bias assessments were conducted. Scholars and institutions continue to wrestle with difficult decisions regarding OA publishing amidst a variety of funding arrangements. More rigorous and robustly reported primary research and follow-up syntheses are needed to equip stakeholders with evidence to make informed choices.

OACA studies could be continuously tracked and reviewed qualitatively, if not quantitatively, in the mode of an emerging method, Living Systematic Reviews (LSRs). The purpose of a LSR is to incorporate new studies as they are published [171]. Further, a LSR could be used to regularly communicate the review status updates with stakeholders as the new evidence is incorporated. Whether or not there is an OACA is a topic of continuing interest and a LSR could support rapidly incorporating new evidence.

## Supporting information

S1 Checklist PRISMA Checklist. This file the PRISMA 2009 checklist with page numbers.
(DOC)

S1 Appendix Search strategy. This file contains the primary database search strategy that was developed in Ovid Medline.
(DOCX)

S1 Dataset Data. This table contains data for all of the included studies.
(CSV)
