# Supplementary material for: Is the open access citation advantage real? A systematic review of the citation of open access and subscription-based articles
Source: PLoS One. 2021 Jun 23;16(6):e0253129. doi: 10.1371/journal.pone.0253129 (PMC8221498; doi:10.1371/journal.pone.0253129)
Supplement: S1 Appendix — This file contains the primary database search strategy that was developed in Ovid Medline. (DOCX) [file pone.0253129.s002.docx]

## S1 Appendix. Search Strategy

Ovid MEDLINE(R) and Epub Ahead of Print, In-Process & Other Non-Indexed Citations, Daily and Versions(R)

1. exp Open Access Publishing/
2. ("open-access" OR (OA NOT osteoarthritis) OR ((open* OR free*) adj1 (access* OR availab*))).tw,kw
3. (journal* OR article* OR publish* OR publication*).tw,kw
4. 2 adj2 3
5. exp Publishing/
6. exp Scholarly Communication/
7. exp Information Dissemination/
8. exp Access to Information/
9. or/5-8
10. 2 and 9
11. 1 or 4 or 10
12. exp Bibliometrics/
13. ((citation* OR cited OR impact) adj2 (metrics OR volume OR high* OR low* OR frequent* OR infrequent* OR advantage* OR disadvantage* OR increas* OR decreas* OR rate*)).tw,kw
14. 12 or 13
15. 11 and 14
16. ..dedup 15
17. limit 16 to yr="2001 -Current"
